# Mr. Brodie and Dr. Hastings on Cysts Connected with the Convex Surface of the Liver

**Published:** 1829-10-01

**Authors:** 


### XLIV.

### CYSTS CONNECTED, AND SUPPOSED TO BE CONNECTED WITH THE CONVEX SURFACE OF THE LIVER.

In the 12th number of the London Medical Gazette is a communication from Mr. Brodie containing two cases of much interest in a practical point of view. A similar case having lately been published by Dr. Hastings, of Worcester, we are induced to collate them, in order that the profession may acquire a truer idea of the affection to which they relate, than either paper of itself would afford. In doing thus, we are merely acting up to a plan we have frequently adopted before, with advantage, as we imagine, to the public.

*Case* 1. In the Spring of 1822, Mr. Brodie was consulted with a medical practitioner, on the case of a young lady 20 years of age, who laboured under a tumour in the right hypochondriac region. Fluctuation was very perceptible in the tumour, which not only lifted up the inferior ribs on that side, but projected distinctly from below them. The tumour had begun to appear, though faintly, a year or two previously, and produced from the first some pain, which increased with its growth, till at length the inconvenience was such that the patient was prevented from sleeping except in a particular position, and from taking any exercise.

The disease advancing in spite of the remedies recommended by Mr. B. and the inconvenience increasing also, he was induced on the 27th of June, 1822, to puncture it with a flat trocar, cautiously introduced below the margin of the ribs. About three pints of watery fluid were evacuated, and care being taken to prevent the ingress of air, and the edges brought together by adhesive plaster, the wound united by the first intention. A bandage was also applied round the upper part of the abdomen, and the patient kept in bed for the first few days after the operation. For two or three weeks she suffered from a very troublesome cough, but as this subsided, she found herself greatly improved, and was soon free from pain, able to lie in any position and to walk as well as other persons. Mr. Brodie saw her in January, 1828, when she continued quite free from any of her former symptoms.

*Case* 2. In August, 1822, a little boy was admitted into St. George's Hospital, under Dr. Chambers, with a tumour presenting itself below the margin of the ribs of the right side, lifting up the ribs also with a distinct fluctuation, and altogether very nearly resembling the tumour which existed in the preceding case. In September, it was determined in consultation to operate, and Mr. Brodie accordingly introduced a flat trocar below the margin of the ribs, and drew off a pint and a half of watery fluid. The boy left the hospital as cured, and has neither been heard of nor seen by Mr. Brodie since. The water evacuated in both cases was clear and colourless, containing no coagulable lymph, and scarcely coagulating at all on the application of heat.

"From the situation of these tumours, I was led to believe," says Mr. Brodie, "that the disease must have been situated in the liver. Cysts are found in the liver containing hydatids, but the circumstance of there being no *debris* of hydatids in the fluid drawn off, and of there having been, in one instance, at least, no return of the disease at the end of five or six years, seem to be at variance with the opinion that such was the nature of the tumour in either of the above cases. Not unfrequently I have noticed in dissection a membranous cyst projecting on the convex surface of the liver, containing a clear, watery fluid. The cysts to which I allude are generally of the size of a marble. The largest which I have chanced to see in the dead body might have contained from one to two ounces of water: but there is no reason why such a cyst should not increase so as to attain a very large size, and I can offer no better explanation than this of the cases which I have now recorded."

We shall presently see that the following case, where dissection, unfortunately, cut the knot, will offer a gratifying corroboration of the truth of Mr. Brodie's conjecture.

*Case* 3.* The patient was a shoemaker, 37 years of age, first seen by Dr. Hastings on the 4th of February, 1820, when he presented the following symptoms. "The abdomen was tumid, hard, and for the most part compressible—there was slight depression extending from about the region of the cæcum, to the epigastrium, where fluctuation was perceived when he was sitting up, but not when lying down—great pain, not constant, in the right hypochondrium, back, and right shoulder, in which he had also an almost constant sense of coldness—when sitting upright, a pretty severe pain across the abdomen at the navel—great dyspnœa, except when lying on the right side; and a distressing cough; expectoration difficult; sputum clear, frothy, of a bluish colour, and salt taste. While out of bed, he sat most with his head leaning on a table, the body inclined forwards, and towards the right side. His sleep was much disturbed by unpleasant dreams and frequent startings. His countenance was expressive of great anxiety; excessive emaciation; pulse 120, not weak; respiration frequent and laborious; tongue of a purplish red; appetite pretty good; considerable thirst. He vomited often in the morning a frothy matter. Bowels costive; fæces dark coloured; urine scanty, with a light red deposit."

He had been engaged in active services as a soldier for seven years, when he returned home in the Summer of 1816, and shortly afterwards became much affected with dyspeptic symptoms, attributed by himself to his employment. In November, 1817, his feet and ankles became œdematous, and the swelling proceeded upwards until it attacked his face. In June, 1818, he was attacked, for the first time, with pain in the right hypochondrium and shoulder, and from that time his symptoms gradually increased. Such were the symptoms, and such the history of this disease, and certainly neither were encouraging. From the perusal one would be tempted to think that both the liver and the heart were implicated, at least the swelling of the face, particularly in the commencement of the disease, are seldom found in dropsy unconnected with cardiac affection. However, to proceed with the case, mercurial frictions were employed at various times with little or no good effect, and afterwards the bowels were merely kept open, and laudanum administered to relieve the pain. The disease marched

* Midland Reporter, No. V. Aug. 1829.

on—the dyspnœa became extreme,—and, the fluctuation, which before was only noticed in the upright posture, was now very distinct in the supine, and accompanied with evident effusion in the lower part of the belly.

On the 11th March, 1829, the operation of tapping was performed by incision with a scalpel, about three inches below the point of the sternum. Eight pounds of fluid were thereby discharged, whilst the patient continued horizontal, and when he sat up to have the bandage adjusted, fluctuation was again perceived in the same spot, and there was an oozing under the strap by which the wound was closed. On the canula being re-introduced, upwards of a pound more of fluid was evacuated. For two days the breathing was relieved by the operation, but on the 13th dyspnœa had returned, the canula was passed once more into the wound, and four pounds of fluid obtained with great relief. After this there was a continual and copious oozing from the wound, the patient progressively sank, and expired early in the morning of the 22d.

*Sectio Cadaveris.* On reflecting the integuments of the thorax and abdomen, and removing the sternum, there appeared in the epigastric region a fine membrane forming large cells, which, when punctured, gave issue to fluid. The membrane in question was situated *between* the peritoneal covering of the liver, and the parietes of the abdomen, and no communication existed between the interior of its cells and the general cavity of the abdomen. It was here that the fluid drawn off by the operation was seen to have been seated. The liver nearly filled the cavity of the abdomen, and pushed up the diaphragm as high as the second ribs, whilst it extended downwards nearly to the pubes. Its peritoneal coat adhered extensively to the diaphragm, and between that coat and the liver itself a vast cyst was formed which contained nearly fourteen pints of bloody serum. The liver in other respects was healthy, and so was the spleen. In the abdomen were about eight pints of fluid, somewhat viscid and tinged with bile, together with a small quantity of purulent matter, though no ulcerating surface could be found. The lungs were small, remarkably collapsed, and adhering pretty strongly to the diaphragm, but otherwise healthy. The cavity of the thorax contained several pints of fluid; the heart was small but healthy.

In the following remarks of Dr. Hastings we perfectly agree, but previously to quoting them, we would venture to hint a difficulty that occurs to us in perusing, or rather understanding the report of the dissection. The cells, we are told, were between the peritoneal covering of the liver and the parietes of the abdomen. Is it meant by this that they were *in* the peritoneal cavity? Unless the peritoneum lining the abdominal parietes were destroyed, they must have been so according to the report, and indeed in accordance with what one would imagine à priori. We will give the remarks of Dr. Hastings without further comment.

"This singular and interesting case seems well worthy consideration, as the subject of it seems to have fallen a victim to a disease, which did not appear to affect the substance of any of the important organs. It is probable that originally the cysts on the convex surface of the liver, which had attained so enormous a size, were not larger than those Mr. Brodie punctured with success; and it is to be presumed, that if the case had been seen sufficiently early for this practice to have been employed, it would have been followed by a like favourable issue.

"The case is also very important, as illustrating the necessity of being very cautious in forming a decided opinion of the existence of organic disease of the liver, where there is very evident enlargement in the neighbourhood of that viscus; for the above case convinces us, that this state of tumefaction may occasionally arise from extraordinary accumulations of fluid in cysts, at the convex surface of the liver, without any disease of that organ.

"This instance also seems satisfactorily to evince, that the cases Mr. B. punctured, were really what he supposed them to be, cysts containing a watery fluid, connected with the liver.

There is also another point to which the attention of the reader should be called; the unnatural situation of the diaphragm, occasioned by the accumulation of fluid at the convex surface of the liver. The cavity of the chest, by this means, was much diminished in size, and hence arose the cough, difficulty of breathing, and disturbed dreams; as likewise the compression of the lungs, and collection of serous fluid in the thorax."

